# Revisiting the rete ovarii

**DOI:** 10.7554/eLife.106648

**Published:** 2025-03-19

**Authors:** Yan Zhang, Hua Zhang

**Affiliations:** 1 State Key Laboratory of Agrobiotechnology, College of Biological Sciences, China Agricultural University Beijing China

**Keywords:** rete ovarii, ovary, proteomics, SNARE complex, fluid secretion, Mouse

## Abstract

Long thought to have little relevance to ovarian physiology, the rete ovarii may have a role in follicular dynamics and reproductive health.

**Related research article** Anbarci DN, McKey J, Levic DS, Bagnat M, Capel B. 2024. Rediscovering the *Rete Ovarii*: a secreting auxiliary structure to the ovary. *eLife* **13**:RP96662. doi: 10.7554/eLife.96662.

The ovary is one of the most complex tissues in the body. This complexity arises from two key aspects: first, as an endocrine organ, the ovary is regulated by both intrinsic secreting factors (Yang et al., 2024) and cyclic hormonal cues (McGee and Hsueh, 2000), with the dynamic interplay between these two signals being vital for reproductive function. Second, while ovarian architecture is primarily shaped by the localization of follicles, which recruit nearby somatic cells on a continuous basis (Liu et al., 2015; Xu et al., 2022), a network of auxiliary structures – such as the oviduct and mesovarium – are also important.

All this complexity means that there are many unsolved mysteries associated with ovarian development and regulation. One such mystery is the rete ovarii, a structure that has long been considered a developmental remnant with little relevance to ovarian physiology (Girsh, 2021). However, recent research using cutting-edge imaging and omics techniques by Blanche Capel of Duke University Medical Center and co-workers has challenged this view by providing new insights into the structure and potential function of the rete ovarii (McKey et al., 2022).

Now, in eLife, Capel and co-workers – including Dilara Anbarci as first author – report how they have used 3D imaging and proteomic analysis to characterize the rete ovarii in unprecedented detail (Anbarci et al., 2024a). The study identified key markers that were found in different regions of the structure, and these markers, along with morphological characteristics, were used to classify the rete ovarii into three distinct regions: the intraovarian rete, the connecting rete, and the extraovarian rete (Figure 1).

**Figure 1.**
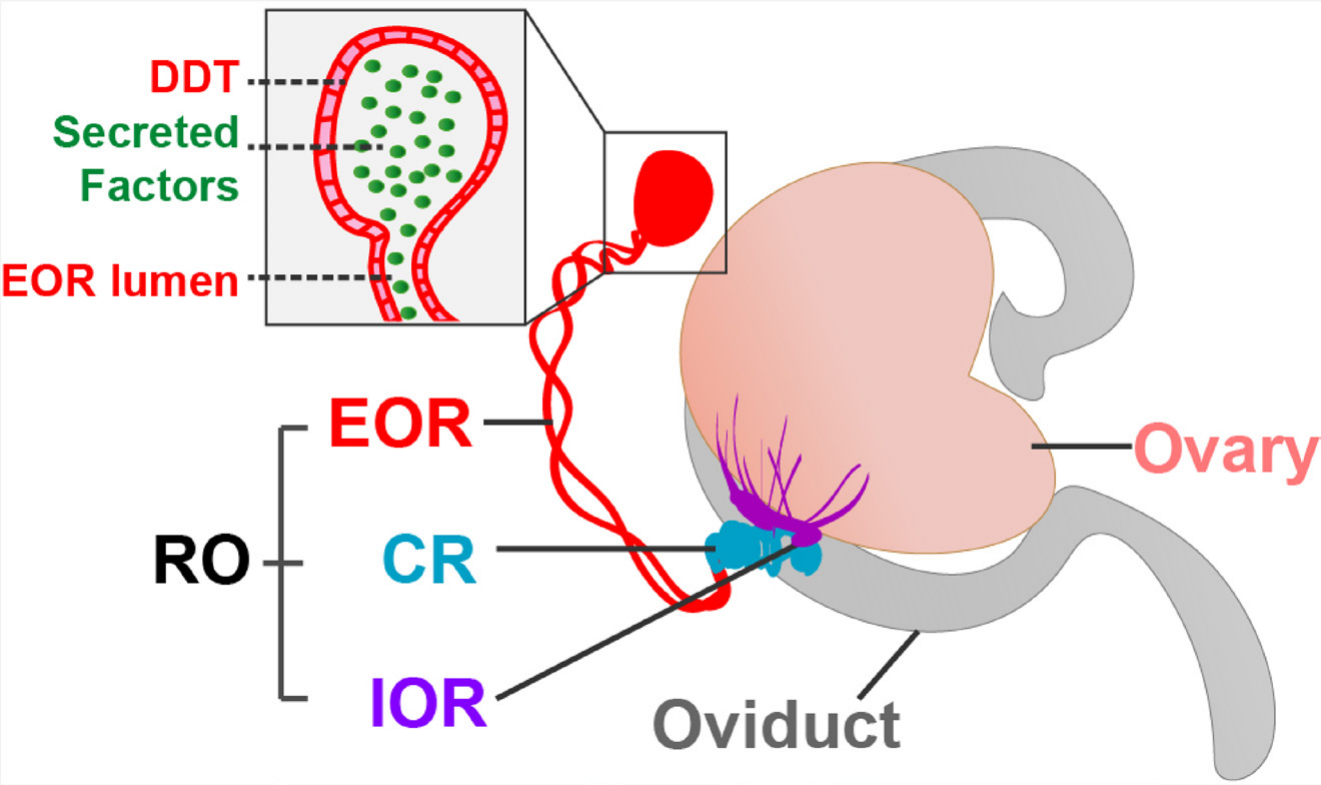
The rete ovarii in neonatal mice. The rete ovarii (RO) is positioned on the dorsal surface of the neonatal mouse ovary (flesh tone), and comprises three distinct regions: the intraovarian rete (IOR; purple); the connecting rete (CR; blue); and the extraovarian rete (EOR; red). The inset highlights secreted factors (green) within the lumen and distal dilated tip (DDT) of the extraovarian rete, underscoring its potential role as a secretory structure.

Adapted from Anbarci et al., 2024a (CC BY 4.0) and McKey et al., 2022 (CC BY 4.0).

The intraovarian rete, located within the ovary, consists of solid cords of squamous epithelial cells. These cords have been linked to early ovarian development and the process by which follicles become mature (folliculogenesis). The connecting rete, as its name suggests, connects the intraovarian rete to the extraovarian rete, gradually acquiring tubular epithelial characteristics closer to the latter. The extraovarian rete is the most developed region, composed of convoluted tubules that terminate in a distal dilated tip. Cells in this region are ciliated and possess a high secretory capacity, suggesting a potential role in fluid transport. Additionally, proteomic analysis reveals secreted proteins, including insulin-like growth factor-binding protein 2 (IGFBP2), which may have a regulatory function in ovarian physiology.

Anbarci et al. propose that the rete ovarii functions as a secretory auxiliary structure to the ovary, and several findings support this groundbreaking hypothesis. Microinjection experiments indicate that secreted factors flow from the lumen of the extraovarian rete toward the ovary, suggesting directional fluid transport. Proteomic analysis reveals that these factors include IGFBP2 and clusterin, which are both involved in ovarian homeostasis. Further, biomolecules essential for vesicular transport and secretion have been identified in the rete ovarii, reinforcing the idea that it has a secretory function.

However, the study has several limitations that remain to be addressed. Anbarci et al. focused mostly on the fetal stage, during which the ovary undergoes rapid morphological changes. While the study provides valuable insights into the embryonic rete ovarii, its role in adult ovarian function remains unclear. Notably, previous studies have demonstrated that even when the mesonephros (the structure from which the rete ovarii is derived) is removed early in development, the formation of follicles in the ovary proceeds normally (Taketo-Hosotani and Sinclair-Thompson, 1987): this suggests that while the rete ovarii may contribute to ovarian physiology, its role during development is likely to be auxiliary rather than essential. Additionally, while the researchers have performed a transcriptomic analysis of the adult rete ovarii in a separate study (Anbarci et al., 2024b), its 3D architecture and functional significance in mature ovaries have yet to be determined.

Rediscovering the rete ovarii challenges the long-standing notion that it is a functionless remnant, and instead positions it as a potentially critical component of ovarian physiology. While the latest results open up new avenues, many questions remain regarding the function of the rete ovarii in adult ovaries, its role in follicular dynamics, and its significance in reproductive health. Further studies are necessary to confirm its secretory functions and explore its role in ovarian biology. As research advances, the rete ovarii may emerge as a key player in reproductive medicine, with potential applications in fertility preservation and ovarian disorder treatment.
